# Prospective clinical study for claudication after endovascular aneurysm repair involving hypogastric artery embolization

**DOI:** 10.1007/s00595-022-02502-x

**Published:** 2022-05-09

**Authors:** Shunya Suzuki, Daijirou Akamatsu, Hitoshi Goto, Takaaki Kakihana, Hirofumi Sugawara, Ken Tsuchida, Yoshitaro Yoshida, Michihisa Umetsu, Takashi Kamei, Michiaki Unno

**Affiliations:** 1 Department of Surgery, Division of Vascular Surgery, Tohoku University Graduate School of Medicine, 1-1, Seiryo-machi, Aoba-ku, Sendai, 980-8574 Japan; 2 Department of Physical Medicine and Rehabilitation, Tohoku University Graduate School of Medicine, Sendai, Japan

**Keywords:** Buttock claudication, Hypogastric artery, Internal iliac artery, Endovascular aneurysm repair, EVAR

## Abstract

**Purpose:**

This prospective study aimed to assess the prognosis of claudication after endovascular aneurysm repair (EVAR) involving hypogastric artery (HGA) embolization.

**Methods:**

Patients who were scheduled to undergo EVAR involving bilateral or unilateral HGA embolization (BHE or UHE, respectively) between May 2017 and January 2019 were included in this study. Patients underwent the walk test preoperatively, one week postoperatively, and monthly thereafter for six months. The presence of claudication and the maximum walking distance (MWD) were recorded. A near-infrared spectroscopy monitor was placed on the buttocks, and the recovery time (RT) was determined. A walking impairment questionnaire (WIQ) was completed to determine subjective symptoms.

**Results:**

Of the 13 patients who completed the protocol, 12 experienced claudication in the 6-min walk test. The MWD was significantly lower at one week postoperatively than preoperatively. The claudication prevalence was significantly higher at five and six months postoperatively after BHE than after UHE. BHE was associated with longer RTs and lower WIQ scores than UHE.

**Conclusions:**

We noted a trend in adverse effects on the gluteal circulation and subjective symptoms ameliorating within six months postoperatively, with more effects being associated with BHE than with UHE. These findings should be used to make decisions concerning management strategies for HGA reconstruction.

**Supplementary Information:**

The online version contains supplementary material available at 10.1007/s00595-022-02502-x.

## Introduction

Surgical treatment is the only approach that can prevent the rupture of abdominal aortic aneurysm (AAA) and iliac artery aneurysm (IAA) [1]. Buttock claudication is a well-recognized complication of intraoperative hypogastric artery (HGA) embolization, with a reported incidence of 8–70%, which can significantly reduce the quality of life [2–13]. Endovascular aneurysm repair (EVAR) is widely used to treat aneurysms [14]. However, the preservation or reconstruction of the HGA through an endovascular approach has anatomical limitations, and the indications for HGA salvage are poorly understood.

Buttock claudication reportedly disappears by 12 months after surgery [4–6], and some studies have reported that its occurrence is not problematic [12]. Bilateral embolization, embolization of the HGA branch, and high preoperative physical activity levels were suggested as risk factors for buttock claudication [2–13]. However, most previous studies on the subject were retrospective and only evaluated information obtained from interviews [2–13]. Although there have been some prospective studies investigating the incidence of buttock claudication and gluteal circulation using walk tests, they did not specify whether or not repeated evaluations were carried out during a given time interval [15–17].

The present prospective study assessed the incidence and prognosis of buttock claudication after HGA embolization. To this end, we conducted a detailed evaluation of gluteal circulation using a walk test and near-infrared spectroscopy (NIRS).

## Methods

### Study design and population

Based on previous studies, the frequency of buttock claudication was assumed to be 40% [2–5], and in this study, it was assumed to increase by 20%. The number of cases required to make a statistically significant difference was set at 44, so we aimed to enroll 50 cases. We recruited all patients over 20 years old who were scheduled to undergo EVAR with HGA embolization for AAA/IAA at our institute between May 2017 and January 2019 for this prospective study. Exclusion criteria were a lack of independence in activities of daily living, a lack of ability to perform moderate-to-vigorous daily physical activity (defined as three metabolic equivalents or more), the presence of active inflammation, the presence of vascular or neurogenic claudication, and undergoing treatment that may affect the activity level (cardiac/cardiovascular surgery, neurosurgery/cerebrovascular surgery, laparotomy, trunk/lower-limb musculoskeletal surgery, or revascularization of lower extremities) during the study period.

This study and all of its protocols were approved by the Ethics Committee of the Tohoku University Graduate School of Medicine and registered at the Information Network Clinical Trial Registry (UMIN-CTR), which was approved by the International Committee of Medical Journal Editors (no. UMIN000025311).

### Surgical protocols

In all patients, contrast-enhanced computed tomography (CECT) was performed preoperatively, and anatomical features (e.g., aneurysm diameter and arterial stenosis) were evaluated. All patients underwent unilateral hypogastric artery embolization (UHE) or bilateral hypogastric artery embolization (BHE), after which a stent graft was placed in the external iliac artery (EIA) as a peripheral landing zone. In cases with isolated iliac aneurysm, the stent graft leg was placed in the common iliac artery/EIA if anatomically acceptable. We defined embolization of the main HGA trunk as “proximal embolization” and separated embolization of the superior gluteal artery (SGA) or inferior gluteal artery (IGA) as “distal embolization.”

### Walk test

The treadmill test (speed, 2.4 km/h; slope, 12%; maximum time, 3 min) and the 6-min walk test [18] were performed preoperatively, within 1 week postoperatively, and then every month until 6 months after surgery. The pain-free walking distance (PWD) and maximum walking distance (MWD) were evaluated from these tests. The MWD ratio ([MWD at each time point − preoperative MWD]/preoperative MWD) was calculated. The treadmill test was designed to increase buttock load by increasing the slope [19] while the 6-min walk test aimed at producing equivalent stress to that experienced during daily living. The two tests were performed at intervals of at least 15 min. We regarded “claudication” to be present if it was noticed at sites where it had not been present before surgery (including the thigh and calf).

### NIRS

During the treadmill test, we placed an NIRS monitor (Hamamatsu Photonics, KK, Hamamatsu, Japan) bilaterally on the hip at the level of the greater trochanter. We measured gluteal oxygenated hemoglobin (oxyHb), deoxygenated Hb (deoxyHb), total Hb, tissue oxygenation index (TOI = oxyHb/totalHb), and recovery time (RT). The RT was defined as the time between the end of the walk test to the intersection of oxyHb and deoxyHb and measured for each limb at up to 300 s after the end of walking in each treadmill test. In cases where RT was > 300 s, we defined RT as 300 s.

### Walking impairment questionnaire (WIQ)

The WIQ [20, 21] was administered during each scheduled walking test, except for that at one week postoperatively, to provide subjective information.

### Endpoints

The primary endpoint was the presence or absence of buttock claudication. The secondary endpoint was changes in the MWD, RT, and WIQ score.

### Statistical analyses

The paired *t*-test was used to analyze paired and normally distributed data, and Wilcoxon’s signed-rank test was used to analyze paired data with a non-normal distribution. Wilcoxon’s rank-sum test was used to analyze unpaired data. Fisher's exact test was used for the contingency table analysis, and Spearman's rank correlation was used to evaluate the bivariate correlation of non-normally distributed data sets. We considered *P* < 0.05 as statistically significant for all analyses and *P* < 0.01 as statistically significant in the case of multiplicity.

## Results

### Patient characteristics

In total, 28 patients were recruited for this study, and 13 were excluded because they were unable to visit the hospital or had begun treatment for other injuries or diseases. Two were not able to complete the follow-up; thus, 13 males (mean age: 78 ± 4.2 years old) were enrolled in the analysis (Table 1). Although we aimed to enroll 50 patients, recruitment was terminated because the period of support from the Japan Society for the Promotion of Science had expired. Preoperative CECT data revealed a bilaterally patent SGA and IGA in all patients, and no significant stenosis was observed in the HGA, SGA, IGA, common femoral artery, or deep femoral artery (DFA) in any cases. EVAR was carried out using a bifurcated stent graft in 10 patients and leg stent graft in 3. Seven patients underwent UHE, and six underwent BHE. Among patients who underwent UHE, four underwent proximal embolization, and three underwent distal embolization. Among patients who underwent BHE, three underwent bilateral proximal embolization and three underwent one-side proximal embolization and other distal embolization. There were no cases of bilateral distal embolization (Table 2). The procedural success rate of HGA embolization and EVAR was 100% for both. No postoperative complications occurred, other than buttock claudication associated with HGA embolization.

**Table 1:** Patient baseline characteristics

	BHE (*n *= 6)	UHE (*n *= 7)	*p* value
Mean age (±SD)	78±3.8yo	79±5.1yo	0.73
Male	6 (100%)	7 (100%)	1
HT	6 (100%)	6 (86%)	1
DL	2 (33%)	1 (14%)	0.56
DM	1 (17%)	2 (29%)	1
CAD	1 (17%)	0	0.46
CVD	0	0	–
COPD	3 (50%)	3(43%)	1
PAD	0	0	–
Complication without buttock claudication			
ABI	0	0	–
Preoperatively			
Left (mean± SE)	1.07±0.04	1.10±0.04	0.50
Right	1.07±0.06	1.13±0.05	0.32
Postoperatively			
Left	1.08±0.05	1.10±0.05	0.42
Right	1.09±0.05	1.12±0.04	0.38

*SD* standard deviation, *HT* Hypertension, *DL* Dyslipidemia, *DM* diabetes mellitus, *CAD* coronary arterial disease, *CVD* cerebrovascular disease, *COPD* chronic obstructive pulmonary disease, *PAD* peripheral arterial disease, *ABI* ankle brachial pressure index, *SE* standard error, *BHE* bilateral hypogastric artery embolization, *UHE* unilateral hypogastric artery embolization

**Table 2:** Anatomical and technical features

	BHE (*n* = 6)	UHE (*n* = 7)	*p* value
Location of Aneurysms			
AAA	2 (33%)	–	0.19
AAA + unilateral CIA	1 (17%)	3 (43%)	0.56
Unilateral CIA	–	2 (29%)	0.46
Bilateral CIA	1 (17%)	–	0.46
Unilateral CIA + bilateral HGA	2 (33%)	–	0.19
Unilateral HGA	–	2 (29%)	0.46
EVAR technique			
Bifurcated	6 (100%)	4 (57%)	0.21
Leg	–	3 (43%)	0.21
Embolization technique			
Proximal	–	4 (57%)	–
Distal	–	3 (43%)	–
Bilateral proximal	3 (50%)	–	–
One side proximal the other distal	3 (50%)	–	–
Embolization device			
Plug	2 (33%)	2 (33%)	1
Coils	1 (17%)	4 (57%)	0.27
Plug and Coils	3 (50%)	1 (17%)	0.27

*AAA* abdominal aortic aneurysm, *HGA* hypogastric aneurysm, *CIA* common iliac aneurysm, *EVAR* endovascular aneurysm repair

### Primary endpoint

Twelve patients (92%) experienced claudication in the 6-min walk test 1 week postoperatively (9 received bifurcated and 3 received leg stent grafts). Eight patients (62%) experienced claudication during the treadmill test 1 week postoperatively (5 received bifurcated and 3 received leg stent grafts).

### Six-minute walk test

**Fig. 1:**
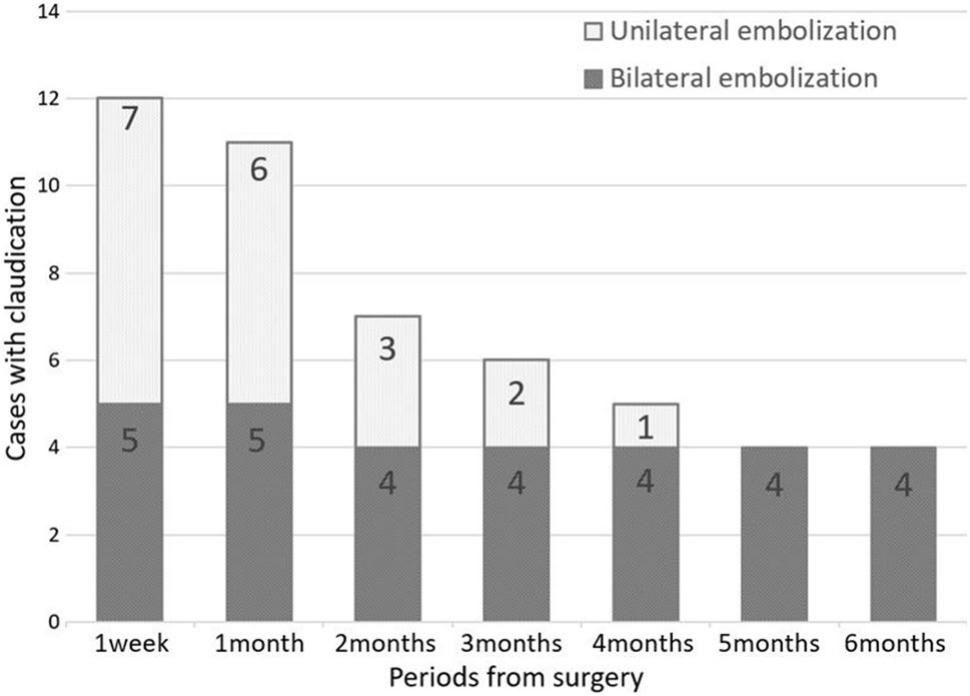
Bar graphs illustrating the incidence of claudication based on the 6-min walk test for patients who underwent bilateral hypogastric artery embolization or unilateral hypogastric artery embolization. 4, 5, and 6 months postoperatively, the incidence of claudication was higher in patients who underwent bilateral hypogastric artery embolization compared to that in patients who underwent unilateral hypogastric artery embolization

Twelve patients experienced claudication one week postoperatively. The sites of pain were the buttocks (*n* = 7), thigh (*n* = 4), and calf (*n* = 5). Three patients with thigh claudication experienced buttock claudication. Four patients experienced only calf claudication, and their postoperative ankle–brachial pressure index (ABI) was not reduced compared with the preoperative ABI. Claudication was observed with reproducibility until it disappeared, regardless of its site. The number of patients experiencing claudication decreased gradually with increasing time after surgery (Fig. 1). All patients with claudication that persisted until five and six months after surgery had undergone BHE. The number of patients with claudication was significantly higher in the BHE group at 5 and 6 months (*p* = 0.02) postoperatively than preoperatively (Fig. 1).

**Fig. 2:**
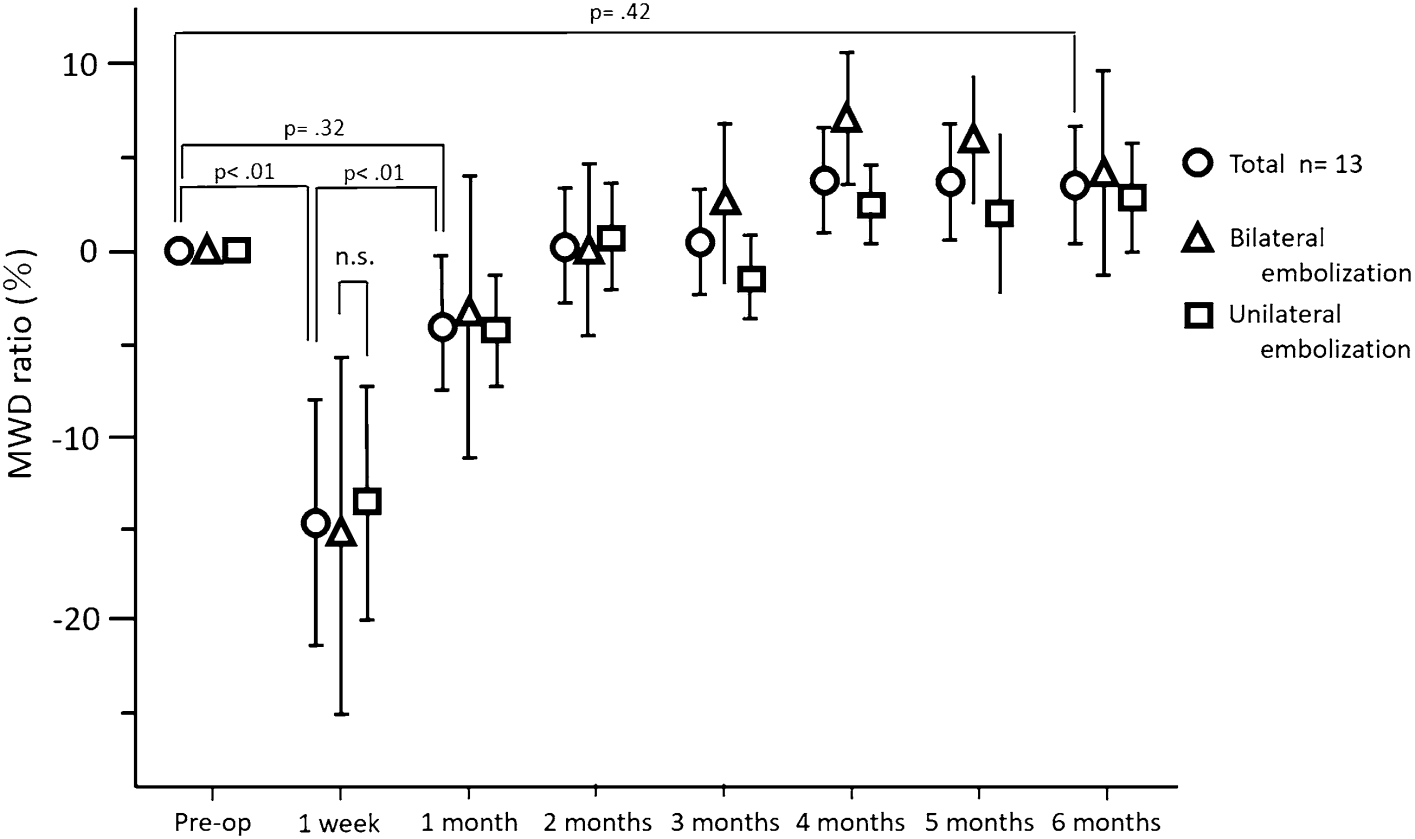
Preoperative and postoperative maximum walking distance (MWD) ratio (%), as determined by the 6-min walk test. The MWD ratio was significantly lower one week postoperatively compared to the baseline. There was no significant difference between the MWD at baseline and after six months postoperatively and between the MWD ratio of patients who underwent bilateral hypogastric artery embolization and that of patients who underwent unilateral hypogastric artery embolization

The MWD ratio one week after surgery was significantly lower than the preoperative value (0 vs. − 0.15 ± 0.20, *p* < 0.01). The MWD ratios were significantly improved at all later time points compared with that at one week postoperatively (Fig. 2). There were no marked differences in the MWD or MWD ratio in relation to BHE or UHE.

There was no significant correlation between the PWD and postoperative duration (*ρ* = 0.23, *p* = 0.10).

### Treadmill test and NIRS

Although 8 patients (62%) experienced claudication during the treadmill test 1 week postoperatively, this number decreased gradually over time (to 7, 6, 5, 4, 5, and 2 patients at 1, 2, 3, 4, 5, and 6 months postoperatively, respectively). The sites of pain were the buttocks (*n* = 7), and calf (*n* = 4). One patient experienced only calf claudication, and his postoperative ABI was not reduced. Claudication was observed with reproducibility until it disappeared, regardless of its site.

**Fig. 3:**
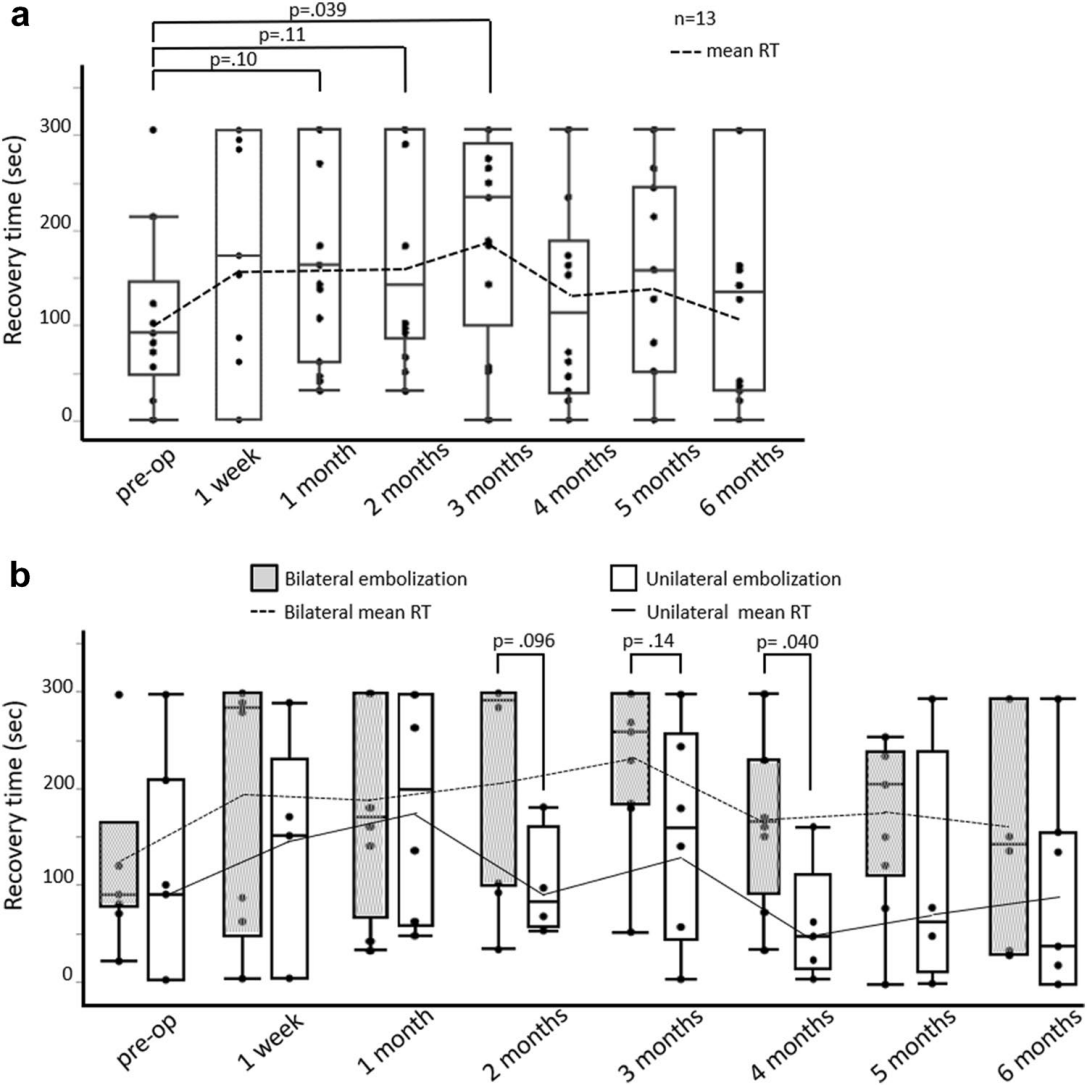
**a** Preoperative and postoperative recovery time. **b** Box plot illustrating the recovery time of patients who underwent bilateral hypogastric artery embolization or unilateral hypogastric artery embolization. Each line graph shows the mean recovery time (RT) at any time points. **a** The RT was significantly longer by three months postoperatively than preoperatively, but this significant difference disappeared 4–6 months postoperatively. **b** The RT tended to be longer by two and four months after bilateral hypogastric artery embolization compared with that after unilateral hypogastric artery embolization

One patient was excluded from the NIRS analysis because of poor-quality image data. The RT 3 months after the surgery was significantly longer than the preoperative value (90 [45, 165] vs. 238 [150, 293], *p* = 0.039; Fig. 3a). However, that significant difference disappeared four–six months after surgery. We found RT to be significantly longer or showing a tendency to be longer among patients who underwent BHE, particularly 2 and 4 months postoperatively, compared with UHE (292.5 [97.5, 300] vs. 80 [53.8, 159], *p* = 0.096; 165 [90, 230] vs. 45 [10, 110] *p* = 0.040; Fig. 3b). There was no significant difference in the TOI between the groups.

### WIQ

**Fig. 4:**
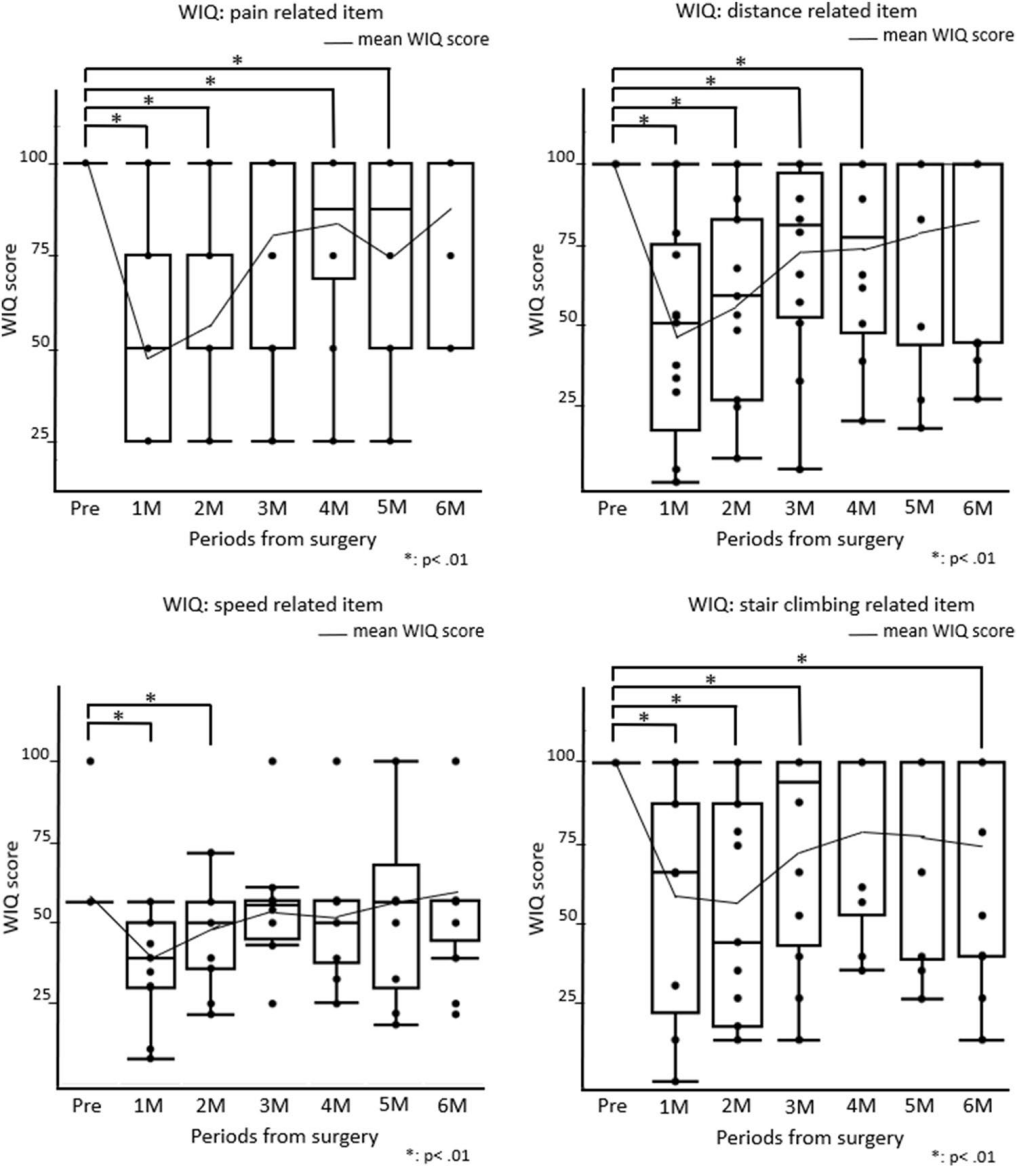
Box plot illustrating the preoperative and postoperative scores on the walking impairment questionnaire (WIQ). Line graphs show the mean WIQ scores at any time points. The WIQ scores were significantly lower in 1, 2, 4, and 5 months postoperatively for pain-related items; 1 and 2 months postoperatively for speed-related items; 1, 2, 3, and 4 months postoperatively for distance-related items; and 1, 2, 3, and 6 months postoperatively for stair climbing-related items than preoperatively

Preoperatively, the WIQ was perfect for all patients, except for speed-related items, as most patients did not make running part of their daily activities. The WIQ scores tended to be lower postoperatively than they had been before surgery (Fig. 4). The WIQ scores, especially in pain- and distance-related items, showed a tendency to ameliorate gradually after surgery (Supplemental Fig. 5).

## Discussion

This prospective study demonstrates the high incidence of reduced walking ability and claudication in the early postoperative period following both UHE and BHE. Although the walking distance returned to the preoperative level within six months postoperatively, patients who underwent BHE reported more persistent subjective symptoms of claudication and a lower quality of life than those who underwent UHE.

Despite the high incidence of claudication associated with HGA embolization, few prospective studies have examined the prognosis of this disorder. This study is unique in providing both subjective and objective evaluations of the natural course of claudication after EVAR involving HGA embolization over a fixed period.

BHE, distal embolization, young age, and high preoperative activities of daily living are reportedly risk factors of buttock claudication [2–13]. The incidence of claudication was 92% in the present study’s cohort, which greatly exceeds previously reported rates [2–13], suggesting that the risk of claudication may be higher than previously considered. We found that there was no significant difference in the initial incidence of claudication between patients undergoing BHE or UHE and between those undergoing proximal or distal embolization. Most previous studies used patient interviews to detect claudication [2–13], whereas we performed a detailed evaluation involving walking tests from one week to six months postoperatively. The protocol of the present study, thus, enabled the detection of claudication associated with HGA embolization beyond the initial postoperative period, which would have been missed in previous studies.

We found subjective symptoms of claudication to be significantly more persistent following BHE than following UHE; this may have been influenced by the development of collateral flow and compensatory mechanisms. A previous study identified DFA as an important source of collateral flow to the ipsilateral HGA [22]. However, the severe symptoms of gluteal ischemia that we observed in patients who underwent BHE suggest that contralateral HGA may be a source of collateral flow. The MWD in the 6-min walk test improved to preoperative levels, regardless of the type of embolization. Thus, patients’ efforts and the development of collateral flow contributed to the recovery of walking distance.

The benefits of NIRS in the evaluation of blood flow in the gluteal muscles have been reported in numerous studies [15–17, 22, 23], and the utility of RT as an indicator of the severity of the peripheral arterial disease has been described [16, 24]. We found that RT (an objective indicator) and WIQ (a subjective indicator) both remained poorer in patients who underwent BHE compared with those who underwent UHE. This might indicate that decreased blood flow that cannot be compensated by the development of collateral vessels occurs following BHE.

Previous studies have not considered infrainguinal claudication as a complication of HGA embolization. However, we found some cases of claudication in the thigh or calf. Gluteal ischemia has been reported to cause lower-limb claudication without a reduced ABI [25]. These cases of claudication were observed with reproducibility and were, thus, regarded as complications of HGA embolization as well as buttock claudication. As the IGA is connected to the DFA [26], the blood flow in the boundary area may depend on either the IGA or DFA. Blood flow to boundary areas via the DFA may increase after HGA embolization to compensate for the decreased blood flow. This may result in decreased flow of the superficial femoral artery, leading to lower-limb claudication. There was no marked difference between the pre- and postoperative rest ABI. If the exercise ABI had been measured, it might have explained the reason behind the occurrence of lower-limb claudication [25, 27, 28].

The inferior mesenteric artery (IMA) and lumbar artery are connected to the HGA [29], and they could be important in collateral flow to the HGA. All patients who underwent EVAR with leg stent graft experienced claudication, and their IMA and lumbar artery were patented. This indicates that claudication can be observed with hypogastric artery embolization regardless of the patency of IMA and lumbar artery. However, we were unable to verify that the patency of the IMA and lumbar artery contributes to the early disappearance of claudication.

The use of two different types of walking tests (treadmill and the 6-min walk test) enabled us to evaluate subjective and objective indicators (i.e., WIQ and RT, respectively). The sites of pain differed between the 6-min and treadmill walk tests, possibly due to the differences in walking speed and the presence of slope.

This study has several limitations that should be acknowledged. First, the sample size was small, which might have prevented us from identifying some statistically significant findings. Second, the follow-up period should be carefully considered. Although we observed recovery of walking distance, recovery of the quality of life and hemodynamics may take longer; therefore, whether or not the test time was appropriate or whether extended data collection would have given different results are unclear at present. However, the extension of the testing time would likely increase the burden on patients. Third, EVAR was carried out using bifurcated and leg stent grafts; therefore, we have not sufficiently evaluated the effects of collateral circulation, such as the IMA and lumbar artery. Fourth, this study used wired NIRS; wireless NIRS may have been more appropriate for avoiding probe misalignment during walking. Although the utility of NIRS for evaluating buttock circulation has been reported [17, 22, 23], the most appropriate method remains debatable. Prolongation of buttock RT was also observed in patients with thigh and calf claudication. It was suggested that the circulation of the gluteal muscles was reduced even in patients with claudication other than in the buttocks [25]. In addition, the RT may have been able to be detected more sensitively by attaching the probe to the sites of pain. Fifth, although this study demonstrates a high incidence of “claudication”, it included not only newly onset postoperative buttock pain but also thigh or calf pain while walking.

## Conclusions

This prospective study demonstrated that the incidence of buttock claudication after EVAR involving HGA embolization may be higher than previously believed. Furthermore, our findings indicate that undergoing BHE is associated with a significantly worse prognosis in terms of claudication, recovery of buttock hemodynamics, and the quality of life up to six months postoperatively than preoperatively. The results of this study will be of interest to clinicians and surgeons and may help inform decisions regarding reconstruction or preservation of the HGA. These findings are also expected to help preserve patients’ activities of daily living and quality of life.

## Supplementary Information

Below is the link to the electronic supplementary material.

Supplementary file1 (PNG 507 KB)
