# Supplementary figures and images for: Prospective clinical study for claudication after endovascular aneurysm repair involving hypogastric artery embolization
Source: Surg Today. 2022 May 9;52(11):1645–52. doi: 10.1007/s00595-022-02502-x (PMC9592672; doi:10.1007/s00595-022-02502-x)

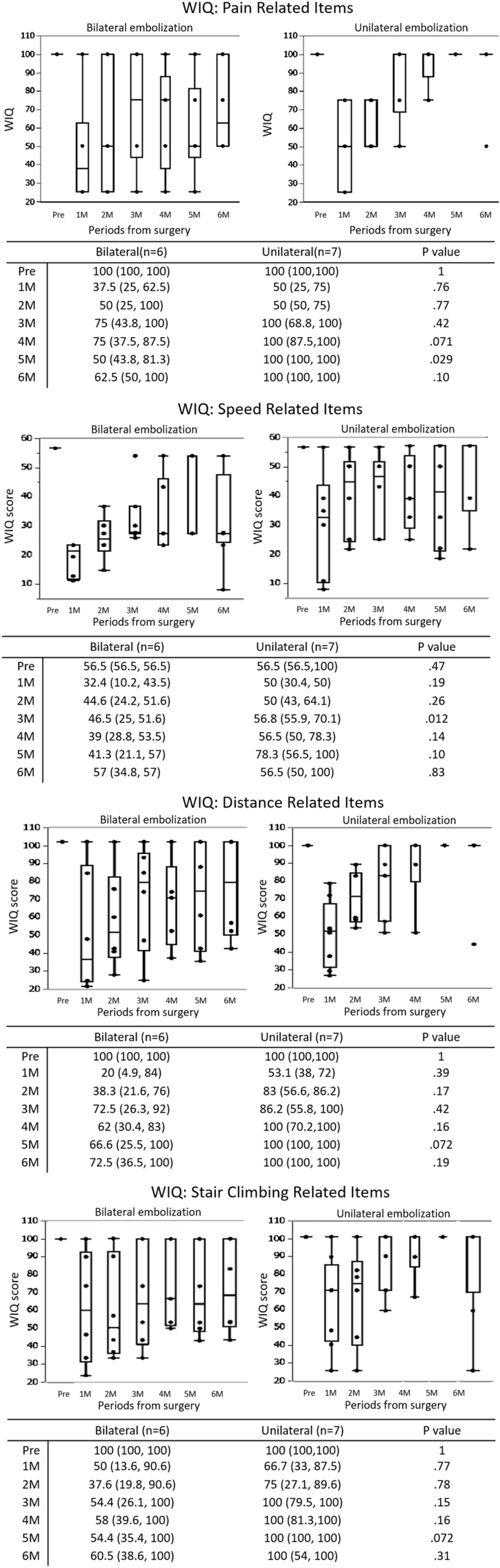

Supplement: Supplementary file 1 — Supplementary file1 (PNG 507 KB) [file 595_2022_2502_MOESM1_ESM.png]
